# Methanotrophic Bacterial Biomass as Potential Mineral Feed Ingredients for Animals

**DOI:** 10.3390/ijerph16152674

**Published:** 2019-07-26

**Authors:** Agnieszka Kuźniar, Karolina Furtak, Kinga Włodarczyk, Zofia Stępniewska, Agnieszka Wolińska

**Affiliations:** 1 Department of Biochemistry and Environmental Chemistry, The John Paul II Catholic University of Lublin, Konstantynów St. 1 I, 20-708 Lublin, Poland; 2 Department of Agriculture Microbiology, Institute of Soil Sciences and Plant Cultivation State Research Institute, Czartoryskich St. 8, 24-100 Puławy, Poland

**Keywords:** methanotrophic bacteria, nutritional values, feed ingredients, fatty acids

## Abstract

Microorganisms play an important role in animal nutrition, as they can be used as a source of food or feed. The aim of the study was to determine the nutritional elements and fatty acids contained in the biomass of methanotrophic bacteria. Four bacterial consortia composed of *Methylocystis* and *Methylosinus* originating from *Sphagnum flexuosum* (Sp1), *S. magellanicum* (Sp2), *S. fallax II* (Sp3), *S. magellanicum IV* (Sp4), and one composed of *Methylocaldum*, *Methylosinus*, and *Methylocystis* that originated from coalbed rock (Sk108) were studied. Nutritional elements were determined using the flame atomic absorption spectroscopy technique after a biomass mineralization stage, whereas the fatty acid content was analyzed with the GC technique. Additionally, the growth of biomass and dynamics of methane consumption were monitored. It was found that the methanotrophic biomass contained high concentrations of K, Mg, and Fe, i.e., approx. 9.6–19.1, 2.2–7.6, and 2.4–6.6 g kg^−1^, respectively. Consequently, the biomass can be viewed as an appropriate feed and/or feed additive for supplementation with macroelements and certain microelements. Moreover, all consortia demonstrated higher content of unsaturated acids than saturated ones. Thus, methanotrophic bacteria seem to be a good solution, in natural supplementation of animal diets.

## 1. Introduction

Aerobic methanotrophs are a unique group of gram-negative bacteria capable of utilization of methane as a sole carbon and energy source [1]. Methanotrophs are present in a wide variety of environments and play an important role in the oxidation of methane in the natural world [2]. There are several reports in the literature database [1,2,3,4,5] that methanotrophic bacteria have been able to inhabit different environmental (sometimes extreme) niches like soils, deserts, landfills, tundra, wetlands, rice paddies, sediments, lakes, and marine environments [1], as well as the atmosphere [3] and coal [4] and salt mines [5].

For more than 30 years, bacteria oxidizing methane have attracted the attention of many researchers and aroused great interest in their industrial applications due to their unique microbiological and metabolic features [2]. Milestones in the study of obligate methanotrophs led to the discovery of their significant potential for applied microbiology, biotechnology, and biochemical engineering, including bioremediation of pollutants (e.g., halogenated hydrocarbons) via co-metabolism by the monooxygenase system (MMOs), biotransformation of diverse organic substrates (e.g., propylene to epoxypropane, production of chiral alcohols), assimilation of methane to mitigate greenhouse effects, and production of commercially relevant compounds, e.g., single cell protein, poly-hydroxybutyrate, astaxanthin [6]. One of the most recent discoveries is the potential of methanotrophic bacteria to compensate for food quantity or food quality limitations in *Daphnia* sp. [7,8]. The results confirm that methane-oxidizing bacteria, possessing sterols and sterol-like compounds, can finally lead to quantitative and qualitative upgrading of phytoplankton diets of *Daphnia* sp. What is more, a series of studies have demonstrated that the methanotrophic community may be a feed for protozoa and myxobacteria, as was confirmed that the composition of the methanotrophic community, in particular type I methanotrophs, changed dramatically during protozoan grazing [9,10,11,12,13,14,15,16,17]. The results obtained by Kiyashko et al. [10] suggest that *Stictochironomus pictulus* can directly feed upon methanotrophic bacteria, because its chironomid tissues contained large amounts of a fatty acid, 16:1 (n − 8), which is specific to the type I methanotroph group (approximately 8% of total fatty acids).

Microorganisms have always been important in basic food processing techniques (e.g., fermentation) and can be used as a source of food or feed [11]. The first commercial product based on the microbial protein (MP) was the Pruteen^®^ from Imperial Chemical Industries. Its production was based on the oxidation of methanol by *Methylophilus methylotrophus* [12]. However, vegetable (soybean) and animal (fishmeal) protein were analyzed for production but were not introduced into the market. The progress in science, especially biotechnology, allows the development of new microbial culture methods, fermentation conditions, and selection of microorganisms [13]. It has succeeded in achieving high production of MP from natural gas utilizing methanotrophic bacteria *Methylococcus capsulatus*, which resulted in the launch of a bacterial protein under the name FeedKind^®^ [12,14]. This product is comparable to traditional protein sources in terms of the amino acid profile and nutrient content. Methanotrophic protein has been used as a protein source for several animal species, including Atlantic salmon, rainbow trout, or pigs [14,15,16,17].

Biotechnology is now playing a major role in the pharmacotherapy of many diseases [18]. Biopharmaceuticals are naturally derived from living cells. As a result, their bio-structure is very complex and their mass is on average 100–1000 times higher than the mass of chemically synthesized pharmaceuticals [19]. Insulin, human growth hormone, blood coagulation factors, and monoclonal antibodies are such commonly used biopharmaceuticals [20]. Bacterial cultures also produce other substances that are important for medicine and cosmetology, e.g., ectoine [21]. The production of MP using methanotrophs yielded 25 g L^−1^ biomass. It contained 310 mg kg^−1^ of iron, 110 mg kg^−1^ of copper, 10–25 g kg^−1^ of phosphorus, 0.2% of magnesium, and 0.8% of potassium [22]. Methanotrophs also have the ability to collect and accumulate rare earth metals [23]. Currently, dietary supplements are produced in most cases by chemical synthesis. The natural solution seems to be an attempt to acquire these nutrients using biotechnological methods. Macronutrients and microelements thus obtained could be highly purified preparations (like other bacterial substances) available to the animal more readily than those derived from chemical syntheses. Bacterial biomass is a potential substitute for ingredients of animal and plant origin such as protein, microelements, and macroelements.

Consequently, the main goals of the study were to analyze the basic nutritional value contained in biomass of methanotrophic bacteria isolated from different environmental niches (endophytes of *Sphagnum* sp., coalbed rock) and to recognize the possibility to obtain fatty acids from methanotrophic bacteria. The important rationale behind undertaking these investigations is also the well-known fact that methanotrophic biomass is not pathogenic [24] and the presence of fatty acids is essential for cholesterol reduction [25,26].

## 2. Materials and Methods

### 2.1. Description of Samples

Biomass originating from different bacterial consortia was studied (Table 1). The Sp1–Sp4 consortium was obtained and described previously by Stępniewska and Kuźniar [27]. It was found that this consortium includes the genera *Methylocystis* and *Methylosinus* [27]. The Sk108 consortium was isolated by Stępniewska et al. [28]. It is composed of three methanotroph strains belonging to genera: *Methylocaldum*, *Methylosinus*, and *Methylocystis.* DNA contents were determined spectrophotometrically (UV-1800, Shimadzu, Kioto, Japan) by measuring UV light absorbance of samples at wavelengths of 260 nm and 280 nm. The measurement was triplicated.

### 2.2. Culture Growth

The bacterial consortia were grown in glass bottles (a capacity of 120 cm^3^) on liquid NMS medium. The medium, proposed in 1970 by Wittenbury [29], is most widely used for growth of methanotrophic bacteria. The NMS medium contained the following components (per L of distilled water): KNO_3_ 1.0 g; MgSO_4_·7H_2_O 1.0 g; CaCl_2_·H_2_O 0.2 g; 3.8% (*w/v*) Fe-EDTA solution 0.1 mL; 0.1% (*w/v*) NaMo·4H_2_O 0.5 mL; KH_2_PO_4_ 26 g; Na_2_HPO_4_·7(H_2_O) 62 g. Additionally, 1 mL of a trace element solution was added (per L of water-distilled solution: FeSO_4_·7H_2_O 500 mg; ZnSO_4_·7H_2_O 400 mg; MnCl_2_·7H_2_O 20 mg; CoCl_2_·6H_2_O 50 mg; NiCl_2_·6H_2_O 10 mg; H_3_BO_3_ (boric acid) 15 mg; EDTA 250 mg). The pH was adjusted to 6.8 using HCl. The glass bottles were incubated for seven days at 30 °C with 180 rpm shaking (Innova 42R, New Brunswick Scientific, Edison, NJ, USA). The growth of methanotrophs was stimulated by supplying CH_4_ (10% *v/v*) for the cultivation. Bacterial multiplication yielded 400 cm^3^ of inoculum of each culture, which served to inoculate the NMS medium in the bioreactors. 4000 cm^3^ of the NMS liquid medium and 400 cm^3^ of the inoculum were placed in each bioreactor (separate for each sample). During the culture, a constant temperature of 30 °C was maintained and air and methane (10% *v/v*) were fed through a sterile filter (0.25 mm diameter). The incubation lasted for 6 days. During this time, the concentration of bacterial cells was determined spectrophotometrically by measurement of absorbance at a wavelength of 600 nm (UV-1800, Shimadzu, Kioto, Japan). The gas phase in the bioreactor was analyzed by a gas chromatograph equipped with three detectors: flame ionization, thermal conductivity, and electron capture (GC 2010, Shimadzu, Kioto, Japan). Each measurement was performed in three replicates.

### 2.3. Concentrations of the Nutrients

The total concentrations of the respective elements were determined (in three replicates) using the flame atomic absorption spectroscopy (FAAS) technique (spectrometer Z-8200 Hitachi, Tokio, Japan) after mineralization of the material (Ethos One, Milestone, Italy). The results were converted to the concentration of each element in 1 kg of liquid bacterial biomass.

### 2.4. Fatty Acid Analysis

Extraction and preparation of fatty acids were performed with the method proposed by Guckert et al. [30] with own modifications. Briefly, an equivalent of 30 mg dry weight of bacterial cells was used for extraction performed at room temperature in 142.5 mL chloroform/methanol/potassium phosphate buffer (1:2.5:0–8 by vol.; 100 mM, pH 7.4) for 3.5 h. During this time, 37.5 mL of chloroform and the same volume of distilled water were added to separate the aqueous (upper) and organic (lower) phases overnight. The next steps were performed according to a procedure developed by Guckert et al. [30]. Samples for PLFA analysis were transesterified by mild alkaline methanolysis with methanolic KOH (methylated) to form PL-FAMEs. Then, the analysis of PL-FAMEs was carried out in the three replicates with the GC technique with an autosampler, split–splitless inlet, Rtx 2330 (Restek company, Bellefonte, PA, USA) column and flame ionization detector (Agilent Technologies, Wilmington, DE, USA). A split ratio of 30:1 was used with hydrogen carrier gas at a 1.2 mL∙min^−1^ constant flow rate. The analysis was carried out in the following conditions: 80–220 °C (10 °C∙min^−1^), 220–230 °C (2 °C∙min^−1^), and 230–260 °C (30 °C∙min^−1^). Fatty acids were identified with the use of standard solutions of fatty acids.

### 2.5. Storage Options

The storage ambient temperature was assessed by keeping the liquid culture at 22 °C for 1 week and incubating in optimal conditions for another one week.

Heat stress was tested by heating the cell suspensions at 80 °C for 10 min, cooling rapidly on ice, plating onto solid medium, and incubating under optimal conditions for 1 week.

Desiccation stress was assessed according to Whittenbury et al. [29] by air-drying suspensions of the consortium on glass slides and then inoculating into the medium after 1 week. Low stress was applied by cooling the cell suspensions at 4 °C for 48 h and then incubating in optimal conditions for 1 week.

Deep freezing was tested by freezing the cell suspensions of the consortium in liquid nitrogen. The culture was kept for 1 year and again incubated in optimal conditions for 1 week.

### 2.6. Statistical Analysis

Statistical analyses were performed using Statistica ver. 10.0 (StatSoft. Inc., Tulsa, OK, USA). Significant differences were calculated with the use of post hoc Tukey’s HSD (honest significant difference) test at a significance level of *p* < 0.05.

## 3. Results

### 3.1. Biomass Growth and Dynamics of Methane Consumption

**Figure 1:**
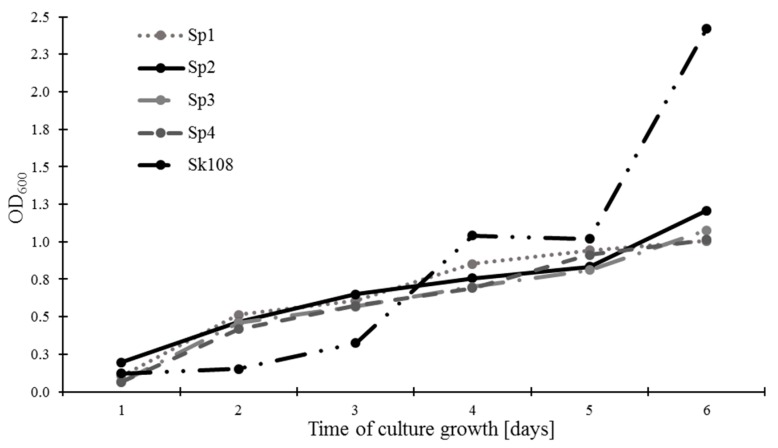
Studied biomass of methanotrophs during culture growth.

The rate of methanotrophic biomass growth expressed as OD_600_ is presented in Figure 1. It was found that, after 6 days of incubation, sample Sk108 was characterized by the highest growth of biomass (2.42), whereas the growth of methanotroph biomass of the Sp1, Sp2, Sp3, and Sp4 combinations exhibited similar levels ranging from 1.01 to 1.21.

**Figure 2:**
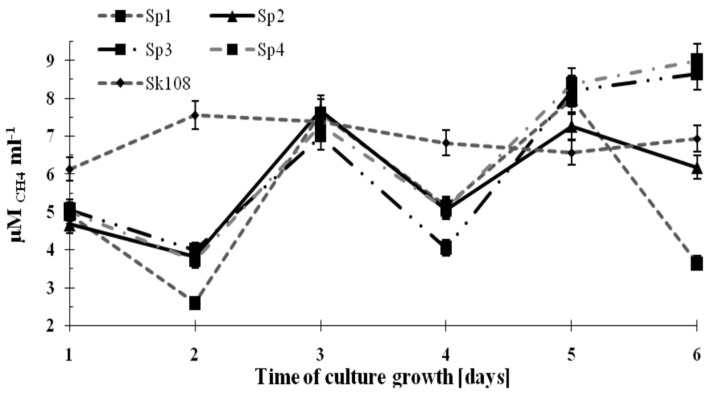
Dynamics of methane consumption by methanotrophs.

The dynamics of methane consumption by the methanotrophs during the incubation time are shown in Figure 2.

It was observed that methane consumption by samples Sp1, Sp2, Sp3, and Sp4 were similar and ranged from 4 to 8 μM CH_4_ mL^−1^. However, on the last incubation day, an increasing trend in CH_4_ consumption up to 8.5 μM CH_4_ mL^−1^ (Sp3) and 9 μM CH_4_ mL^−1^ (Sp4) was noted. Methane consumption in sample Sk108 over the 6 incubation days was at an almost constant level (6.14–7.57 μM CH_4_ mL^−1^).

The fluctuations in the values of methane consumption by the studied consortia are the consequence of supplementing only one source of carbon and energy (CH_4_). Supplementation was applied every 2 days (Figure 2). These fluctuations did not affect the growth of the methanotrophic biomass.

### 3.2. Fatty Acids in the Methanotrophic Biomass

The results of the total fatty acid content of the biomass of the analyzed consortia are presented in Table 2.

The concentration of saturated acids oscillated from 4.29% to 32.85%. The analysis of unsaturated fatty acids showed the presence of omega-5 (1.36%–3.01%), omega-6 (0.93%), omega-7 (1.34%–19.75%), and omega-9 (0.5%–92.48). The concentration of unsaturated fatty acids was similar in biomass Sp1, where it amounted to 95.21%, and in Sp2 (94.23%). In turn, lower levels were found in biomass Sp3 (82.99%), Sp4 (81.56%), and Sk108 (67%). The presence of a small amount of unidentified acids was only reported in the consortia of Sp2 and Sp3 (1.48% and 1.97%, respectively). It was found that all consortia had higher contents of unsaturated acids than saturated acids.

### 3.3. Macro- and Micronutrient Concentrations in the Methanotrophic Biomass

The concentrations of macronutrients (K, Mg, Ca, Fe, and Na) in the methanotrophic biomass are presented in Table 3. The highest content in the microbial biomass was recorded in the case of K (9.592–19.100 g·kg^−1^), followed by Mg (2.243–7.594 g·kg^−1^), Fe (2.436–6.594 g·kg^−1^), and Ca (2.008–3.274 g·kg^−1^).

The lowest macronutrient content was found in respect to Na (1.331–1.910 g·kg^−1^). Regardless of the element type, the highest potential for macronutrients accumulation was exhibited by methanotrophs consortium Sk108, followed by consortium Sp3. Considerable statistically significant differences were noted in the K and Mg content.

The concentrations of micronutrients (Zn, Cu, Mn, and Cr were determined in the methanotrophic biomass (Table 4) analogically to the macronutrient contents.

The micronutrient content was below 1 g·kg^−1^. Mn and Cu were present in the highest concentrations, i.e., 0.267–0.720 g·kg^−1^ and 0.175–0.476 g·kg^−1^, respectively. The content of Zn ranged from 0.087 to 0.142 g·kg^−1^, whilst Cr was found to be the least represented micronutrient (0.009–0.166 g·kg^−1^) Furthermore, no Cr was detected in two samples of bacterial biomass (Sp1 and Sk108) (Table 4). The differences in the content of the microelements were statistically significant.

### 3.4. Dietary Requirements for Nutrients in Different Animals

Table 5 presents daily (estimated) supplementation of macroelements originating from methanotrophic bacterial biomass in animals.

The addition of 1 kg of bacterial biomass to swine feed could ensure supplementation of K and Mg above the recommended daily intake (RDI; 191%–365% for K and 182%–617% for Mg). Addition of the analyzed biomass (1 kg) to swine feed could cover only 11%–17% and 51%–62% of their RDI Ca and Na demand, respectively.

In the case of dog feed, addition of 1 kg of the bacterial biomass originating from Sk108 could cover 27% and 6.4% of the RDI Mg and K demand, respectively.

In order to supplement Ca or Na, the amount of added biomass should be increased, as 1 kg of the bacterial biomass does not cover even 2% of dog’s daily needs for these minerals (Table 5). Nevertheless, addition of 1 kg of bacterial biomass originating from sample Sk108 to the feed can help supplement the amount of K (by 26% of the RDI) and Mg (by 45% of the RDI) in the cow’s diet. This amount of bacterial biomass will also provide 14% and 5% of the daily Na and Ca demand, respectively (Table 5).

The amount of Mg contained in 1 kg of the bacterial biomass is a multiple of the daily requirement for this element in broiler chickens. This amount of biomass contains up to 159% (Sk108) of the broiler chicken’s daily potassium demand and up to 40% (Sp1) of the daily Na dose. 

The macroelements content in 1 kg of the consortium Sk108 bacterial biomass could provide 108% of the daily Fe dose for dogs, 8.24% for broiler chickens, 5.36% for swine, and 3.62% for cows. The largest percentage of supplementation of all macroelements could be provided by the addition of the biomass of consortium Sk108 bacteria (Table 5).

The highest percentage of copper RDI could be provided by addition of 1 g of the biomass of the studied consortium for dogs (from 23.12% by Sp2 to 62.68% by Sp1) (Table 6).

An addition of 1 g of the bacterial biomass from the Sp3 consortium to feed could provide from 0.29% of the daily Mn dose for cows to 189.5% for dogs. The Zn content in the bacterial biomass (1 g) would represent from 0.03% (Sp2 and Sp4) of daily requirement for this element for swine to 2.08% (Sp3) for dogs. In the case of Mn, the highest percentage of daily consumption could be provided by addition of 1 g of the biomass of the studied consortium for dogs: 70.41% by Sk108, 104.04% by Sp4, 126.12% by Sp1, 146.41% by Sp2 and 189.55% by Sp3.

### 3.5. Storage Options for Methanotrophic Biomass

The methanotrophic activity in optimal conditions of culture (30 °C, aerobic condition) was in the range of 1.364–2.123 µM CH_4_ mL^−1^d^−1^ (Table 7).

The methane oxidation rate at ambient temperature was comparable to the CH_4_ consumption in the studied consortium grown in optimal culture conditions, i.e., from 1.429 to 2.127 µM CH_4_ mL^−1^d^−1^. In turn, the methanotrophic activity of the consortium significantly decreased from 0.075 to 0.147 µM CH_4_ mL^−1^d^−1^ at lower temperature (4 °C). During the thermal treatment (80 °C for 10 min), the highest methane consumption was observed in consortia Sp1 and Sp4, i.e., 2.173 and 2.013 µM CH_4_ mL^−1^d^−1^, respectively.

After direct deep freezing, the methanotrophic activity of the studied consortium was lowered. The CH_4_ consumption value ranged from 0.025 to 0.819 µM CH_4_ mL^−1^d^−1^. The viability of the studied consortium exhibited a varied level.

The experimental conditions, i.e., ambient and low temperature caused slight changes in culture viability, which ranged from 74.125 to 89.008 µM CH_4_ mL^−1^d^−1^. During storage in the drying conditions, the relative percentage of viable bacterial consortia Sk108, Sp3, and Sp4 decreased dramatically, compared to optimal conditions of consortium growth. Survival during this storage type in the Sp1 and Sp2 biomass was at a level of 50%–55%. The highest cell viability reduction was noted during the thermal treatment. Survival during this storage had values from 5.231% to 33.586%. The LIVE/DEAD staining test demonstrated 14.957%–23.595% viability of the cell consortium after deep freezing.

## 4. Discussion

Given the literature data regarding the metabolism of methanotrophs, it can be concluded that their biomass contains large quantities of Ca [35], Cu [36], and P [17,37]. These elements are also cofactors of the major enzymes involved in the biological oxidation of methane: methane monooxygenase (pMMO and sMMO), methanol dehydrogenase, and formate dehydrogenase. The results of our research demonstrated high contents of K, Mg, and Fe in the methanotrophic bacterial biomass (Table 3), i.e., approx. 9.6–19.1, 2.2–7.6, and 2.4–6.6 g kg^−1^, respectively. The Ca and Na contents were lower, i.e., in the range of 2–3.3 and 1.3–1.9 g kg^−1^, respectively (Table 3). In turn, the Cu concentration of the biomass was much lower than assumed (0.1–0.4 g kg^−1^). However, our results suggest that the biomass of methanotrophic bacteria can be an appropriate feed and feed additive for supplementation with macroelements (K, Mg, Ca, Na, Fe) and certain microelements (Cu, Mn). In addition, as shown by other data, the biomass of methanotrophic bacteria is also rich in vitamins B (niacin, riboflavin) [14], which is an additional advantage from the dietary point of view. The methanotrophic bacterial biomass can be added to feed in the form of freeze-dried feeding stuffs.

Some methanotrophic bacteria e.g., *Methylomonas methanica* and *Methylococcus capsulatus* are also used for the production of single cell protein added to animal feeds [38,39]. In the production of single cell proteins (SCP) using *M. capsulatus,* biomass containing 310 mg kg^−1^ of Fe and 110 mg kg^−1^ of Cu was obtained [22]. However, it should be underlined that these bacteria (*M. capsulatus*) were grown in an industrial culture of mixed *M. capsulatus* Bath with *Bacillus brevis* DB4, *Bacillus firmuj* DB5, and *Alcaligenes acidovorans* DB3; therefore, it cannot be claimed that the content of Cu and Fe originated only from the biomass of *M. capsulatus*. The production of single cell proteins using methanotrophs ensures an amino acid composition comparable to that of fishmeal and soybean meal [14,40]. In 2005, the EU approved protein derived from bacterial biomass growing on natural gas for use in pig, calf, and salmon diets [41]. The process of SCP production based on *M. capsulatus* was designed to fine-tune proteins for salmon, chickens, and pigs. However, this proved to be uneconomic because of the high price of natural gas [42,43]. Therefore, SCP production based on methane from natural sources (e.g., sediments, landfills) is a promising research trend. Aas et al. [15] have shown that the addition of biomass of methanotrophic bacteria (36%) to the salmon diet increased the retention of N in the fish body (resulting in higher retention of N and energy). An increase in salmon body weight was observed as well [14,44].

Yet, it is important that methanotrophic bacterial biomass cannot be used in human nutrition due to the excessive nucleic acid content. An option is to partially remove and decompose nucleic acids from biomass [11]. Nevertheless, it has been found that methanotrophic microorganisms can be food for protozoa and mucous bacteria [9]. In the case of *Daphnia*, a community of methanotrophs leads to quantitative and qualitative improvement in the diet [7,8]. A similar finding has also been demonstrated in *Stictochironomus pictulus*, which can directly feed on methanotrophic bacteria [10].

Unsaturated acids from the omega-3 (e.g., α–linolenic acid) and omega-6 (e.g., linoleic acid and arachidonic acid) groups were shown to exert health effects on animals and should therefore be delivered to the body along with food. The presence of these acids in the diet reduces blood cholesterol levels, increases immunity, and is essential for normal brain function. Our analysis of unsaturated fatty acids proved that omega-5, omega-6, omega-7, and omega-9 acids are present in the consortia of methanotrophic bacteria. The presence of high levels of unsaturated fatty acids in the Sp1 and Sp2 consortia indicates that they can potentially be used to obtain these acids in the biotechnology industry. Unsaturated acids produced by bacteria could be used in animal feed and medicinal products. From this point of view, the biomass addition to animal feed and supplementation of unsaturated acids are more important for animal health than saturated acids. The appropriate concentration of unsaturated fatty acids has an impact on the quality of meat, milk, or eggs obtained from farmed animals. The supply of these acids with food is also directly reflected in the fatty acid profile that will be present in animal organisms [45]. Bacterial fatty acids could therefore be used in pharmaceuticals, nutraceuticals, and food. Furthermore, methanotrophs are particularly effective in the production of lipids, as their PLFAs represent more than 30% by weight [46]. A patent for the production of fatty acids from methanotrophs to reduce cholesterol [47] has already been developed.

Furthermore, several studies have shown that the use of methanotrophic biomass can improve meat quality in monogastric farm animals (swine, dairy cattle, broiler chicken) [48,49,50]. No mortality or negative effects of methane prophyroid biomass on clinical health of tested animals or changes in food ducts have been observed. Methanotrophic biomass is well tolerated by all tested animal species. Importantly, these bacteria are not pathogenic [37,44,48]. In addition, such protein does not contain toxins and GMOs, as it is a completely natural product and can be used as a protein feed ingredient [38,51].

The nucleic acid concentration is an important feature of biomass that can be used as a diet, as uric acid is the product of purine metabolism, whose high concentration contributes to development and intensification of the symptoms of gout. The maximum permitted daily dose of nucleic acids for an adult human is 4 g [52]. That is why it is so important to determine the concentration of nucleic acids in the cultured biomass. In our study, the DNA content in methanotrophic biomass (Table 2) was significantly lower than the maximum permitted dose, i.e., it ranged from 26.47 to 43.55 ng/µL. The calculated DNA concentration was in agreement with values presented by Lau et al. [53], who also measured the DNA content in *Sphagnum* and samples taken from peat soils. This additionally confirms the safety of methanotrophic biomass for application as a feed ingredient for animals.

Last but not the least, an important highlight is the fact that methanotrophic bacteria belong to the mesophilic group of microorganisms, which means that the temperature optimum for their growth is between 20 and 37 °C [54], whereas the minimum and maximum are 10–30 °C and 35–50 °C, respectively [55]. Temperature is an extremely important aspect of biomass storage conditions regulating methanotrophic activity and mineral content. Our study evidenced that the optimal temperature for biomass storage should oscillate between 10 and 30 °C, which means that an easy way of providing methanotrophic biomass storage involves providing room temperature. Nevertheless, sterile conditions must be maintained, because, a majority of microorganisms found in nature are unfortunately included in the group of mesophile. Consequently, there are both saprophytic and pathogenic organisms in this group [56].

Another unquestionable benefit of using methanotrophic bacteria as an additive to feed is the possibility of using mineral growth medium [5] guaranteeing the absence of other microorganisms-e.g., from the *Enterobacteriaceae* family, which may potentially be harmful in nutrition. Additionally, through the application of methanotrophic biomass to feed, it is possible to avoid any GMO modifications and use of any genetic engineering to increase the synthesis of the vitamin due to the high plasticity of methanotroph metabolism. What is more, the product proposed by us comprises natural, non-toxic strains, thus eliminating a conflict with society resulting from the lack of acceptance of technologies based on modified organisms.

## 5. Conclusions

In this paper, we have presented the possibility of use of methanotrophic bacterial biomass as potential mineral feed ingredients dedicated to different animals. It was shown that all biomass obtained from the studied consortium had higher contents of unsaturated acids than saturated acids. Moreover, the analyzed biomass displayed high potential for accumulation of macronutrients and micronutrients. We concluded that the conditions of cell consortium storage are very important. The low temperature (4 °C) was proved to be the most suitable for biomass storage, while ambient temperature contributed to high bacterial cell survival. Currently, modern biotechnology is looking for microorganisms with unique metabolic characteristics (especially having health-enhancing properties) that are easy to grow and use a cheap source of carbon and energy. In this context, methanotrophic bacteria seem to be a pioneering solution and a new perspective, e.g., in supplementing animal diets, or as a new source of substances with a beneficial effect on the development and growth of animals.

## Figures and Tables

**Table 1:** List of samples with DNA content (± SD).

Symbols	Explanations	DNA Concentration (ng·µL^−1^)
Sp1	Consortium of methanotrophs from *Sphagnum flexuosum*	26.47 ± 0.04
Sp2	Consortium of methanotrophs from *Sphagnum magellanicum* II	32.22 ± 0.05
Sp3	Consortium of methanotrophs from *Sphagnum fallax*	29.74 ± 0.11
Sp4	Consortium of methanotrophs from *Sphagnum magellanicum* IV	39.85 ± 0.16
Sk108	Consortium of methanotrophs from coalbed rock	43.55 ± 0.12

**Table 2:** Contents of fatty acids in methanotrophic bacteria (nd–not detected).

Type of Biomass	Saturated Fatty Acids				Unsaturated Fatty Acids					Unknown Fatty Acids
	C14:0	C15:0	C16:0	C18:0	C16:1ω5	C16:1ω7	C16:1ω9	C18:1ω9	C18:2ω6	
Sp1	1.53	nd	nd	3.26	nd	nd	2.73	92.48	nd	-
Sp2	nd	nd	3.52	0.77	nd	1.34	0.50	92.39	nd	1.48
Sp3	0.90	0.15	12.31	1.68	1.36	19.75	22.91	38.04	0.93	1.97
Sp4	nd	nd	11.50	7.19	3.01	6.80	10.63	60.87	nd	-
Sk108	27.21	29.65	43.14	1.09	nd	nd	nd	67.15	nd	-

**Table 3:** Contents of macronutrients in methanotrophic biomass.

Biomass	Macronutrients				
	K [g·kg^−1^]	Mg [g·kg^−1^]	Ca [g·kg^−1^]	Fe [g·kg^−1^]	Na [g·kg^−1^]
Sp1	10.031 ± 0.01 ^d^	6.086 ± 0.006 ^b^	2.664 ± 0.001 ^c^	3.286 ± 0.001 ^c^	1.910 ± 0.0002 ^a^
Sp2	10.316 ± 0.01 ^c^	2.243 ± 0.002 ^e^	2.618 ± 0.001 ^c^	2.436 ± 0.001 ^d^	1.562 ± 0.0006 ^b^
Sp3	12.556 ± 0.01 ^b^	3.274 ± 0.003 ^c^	2.850 ± 0.001 ^b^	4.408 ± 0.0003 ^b^	1.331 ± 0.001 ^d^
Sp4	9.592 ± 0.01 ^e^	2.274 ± 0.001 ^d^	2.008 ± 0.001 ^d^	2.480 ± 0.001 ^d^	1.373 ± 0.0003 ^c,d^
Sk108	19.100 ± 0.02 ^a^	7.594 ± 0.002 ^a^	3.274 ± 0.001 ^a^	6.594 ± 0.002 ^a^	1.422 ± 0.001 ^c^

The presented values are the average of three replicates (*n* = 3); ± standard deviation (SD); means marked with different letters (a–e) are significantly different at *p* < 0.05 (*n* = 3) as shown by Tukey’s HSD test.

**Table 4:** Contents of micronutrients in methanotrophic biomass.

Biomass	Micronutrients			
	Zn [g·kg^−1^]	Cu [g·kg^−1^]	Mn [g·kg^−1^]	Cr [g·kg^−1^]
Sp1	0.108 ± 0.006 ^c^	0.476 ± 0.021 ^a^	0.479 ± 0.022 ^b,c^	<0.005 ^c^
Sp2	0.087 ± 0.008 ^d^	0.175 ± 0.033 ^c^	0.556 ± 0.042 ^b^	0.009 ± 0.035 ^b^
Sp3	0.142 ± 0.004 ^a^	0.279 ± 0.019 ^b^	0.720 ± 0.081 ^a^	0.166 ± 0.096 ^a^
Sp4	0.096 ± 0.002 ^c,d^	0.185 ± 0.033 ^c^	0.395 ± 0.001 ^c^	0.047 ± 0.012 ^a,b^
Sk108	0.126 ± 0.003 ^b^	0.416 ± 0.029 ^a^	0.267 ± 0.017 ^d^	<0.005 ^c^

The values are the average of three replicates (*n* = 3); ± standard deviation (SD); means marked with different letters (a–c) are significantly different at *p* < 0.05 (*n* = 3) as shown by Tukey’s HSD test.

**Table 5:** Estimated daily supplementation [%] of macronutrients for selected animals with the use of methanotrophic bacterial biomass originating from different consortia. Each value is calculated for 1 kg of bacterial biomass (according to references [31,32,33,34]).

Sample	K	Mg	Ca	Na	Fe
**Swine; 1 kg Bacterial Biomass**					
Sp1	191.80	494.80	14.14	62.01	2.67
Sp2	197.25	182.36	13.90	50.71	1.98
Sp3	240.08	266.18	15.13	43.21	3.58
Sp4	183.40	184.88	10.66	44.58	2.02
Sk108	365.20	617.40	17.38	46.17	5.36
**Dog; 1 kg Bacterial Biomass**					
Sp1	3.34	22.21	0.39	1.91	53.87
Sp2	3.44	8.19	0.39	1.56	39.93
Sp3	4.19	11.95	0.42	1.33	72.26
Sp4	3.20	8.30	0.30	1.37	40.66
Sk108	6.37	27.72	0.48	1.42	108.10
**Dairy Cattle; 1 kg Bacterial Biomass**					
Sp1	13.78	36.23	4.23	13.64	1.81
Sp2	14.17	13.35	4.16	11.16	1.34
Sp3	17.25	19.49	4.52	9.51	2.42
Sp4	13.18	13.54	3.19	9.81	1.36
Sk108	26.24	45.20	5.20	10.16	3.62
**Broiler Chicken; 1 Kg Bacterial Biomass**					
Sp1	83.59	1014.33	8.33	39.79	4.11
Sp2	85.97	373.83	8.18	32.54	3.05
Sp3	104.63	545.67	8.91	27.73	5.51
Sp4	79.93	379.00	6.28	28.60	3.10
Sk108	159.17	1265.67	10.23	29.63	8.24

**Table 6:** Estimated daily supplementation [%] of microelements for selected animals with the use of methanotrophic bacterial biomass originating from different consortia. Each value is calculated for 1 g of bacterial biomass (according to references [31,32,33,34]).

Sample	Zn	Cu	Mn
**Swine; 1 g Bacterial Biomass**			
Sp1	0.07	5.16	7.79
Sp2	0.06	1.90	9.05
Sp3	0.09	3.03	11.71
Sp4	0.06	2.01	6.43
Sk108	0.08	4.51	4.35
**Dog; 1 g Bacterial Biomass**			
Sp1	1.58	62.68	126.12
Sp2	1.28	23.12	146.41
Sp3	2.08	36.75	189.55
Sp4	1.41	24.37	104.04
Sk108	1.85	54.76	70.41
**Dairy cattle; 1 g Bacterial Biomass**			
Sp1	0.04	0.26	0.19
Sp2	0.03	0.10	0.22
Sp3	0.05	0.15	0.29
Sp4	0.03	0.10	0.16
Sk108	0.04	0.23	0.11
**Broiler Chicken; 1 g Bacterial Biomass**			
Sp1	0.27	5.95	0.80
Sp2	0.22	2.20	0.93
Sp3	0.36	3.49	1.20
Sp4	0.24	2.32	0.66
Sk108	0.32	5.20	0.45

**Table 7:** Methanotrophic activity and viability of the analyzed biomass in the storage options.

Storage Options	Methanotrophic Activity [µM CH_4_ mL^−1^d^−1^]					Viability [%]				
	Sp1	Sp2	Sp3	Sp4	Sk108	Sp1	Sp2	Sp3	Sp4	Sk108
Ambient temperature	1841 ^c,v^	2127 ^a,u^	1462 ^d,v^	1947 ^b,c,u^	1429 ^d,u^	82,145 ^c,v^	75,250 ^d,v^	89,008 ^a,v^	85,131 ^b,v^	86,705 ^a,b,v^
Dry condition	0.147 ^a,y^	0.091 ^b,x^	0.138 ^a,x^	0.144 ^a,x^	0.075 ^b,x^	55,031 ^a,w^	50,230 ^b,w^	15,085 ^c,y^	10,833 ^d,z^	0.909 ^e,z^
Low temperature	1146 ^b,w^	1546 ^a,v^	0.954 ^c,w^	0.871 ^c,v^	0.431 ^d,w^	82,462 ^a,b,v^	74,125 ^d,v^	82,014 ^b,c,w^	80,146 ^c,w^	84,264 ^a,w^
Heat temperature	2173 ^a,u^	0.488 ^d,w^	0.974 ^b,w^	2013 ^a,u^	0.714 ^c,v^	5,231 ^e,y^	11,565 ^b,y^	6894 ^d,z^	33,586 ^a,x^	8,542 ^c,y^
Deep freezing	0.819 ^a,x^	0.072 ^b,c,x^	0.034 ^b,c,x^	0.025 ^c,x^	0.098 ^b,x^	12,159 ^e,x^	14,957 ^d,x^	23,595 ^b,x^	24,959 ^a,y^	17,507 ^c,x^
Optimal condition of culture	1954 ^a,b,uv^	2123 ^a,u^	1756 ^b,u^	1994 ^a,b,u^	1364 ^c,u^	96,354 ^b,c,u^	97,365 ^a,b,u^	95,654 ^c,u^	96,468 ^b,c,u^	98,654 ^a,u^

Different letters indicate significant differences as shown by Tukey’s HSD test at *p* < 0.05. Values with different letters “a–e” (in the rows) indicate significant difference for different biomass at the same storage options, and “u–z” (in the columns) indicate significant difference for the same biomass in different storage options; both at *p* ≤ 0.05 in Tukey’s HSD test.
